# Ketamine–Propofol Coadministration for Induction and Infusion Maintenance in Anesthetized Dogs: Effects on Electroencephalography and Antinociception

**DOI:** 10.3390/ani13213391

**Published:** 2023-11-01

**Authors:** Jeff C. Ko, Carla Murillo, Ann B. Weil, Matthias Kreuzer, George E. Moore

**Affiliations:** 1 College of Veterinary Medicine, Purdue University, West Lafayette, IN 47907, USA; murilloc@purdue.edu (C.M.); aweil@purdue.edu (A.B.W.); gemoore@purdue.edu (G.E.M.); 2 School of Medicine, Technical University of Munich, 80333 Munich, Germany; m.kreuzer@tum.de

**Keywords:** ketamine, propofol, constant rate infusion, dogs, EEG, electric stimulation, antinociception, cardiorespiratory, biphasic, behavior, tongue flicking, muscle activity, ketofol

## Abstract

**Simple Summary:**

The coadministration of ketamine and propofol (ketofol) via constant rate infusion (CRI) was studied as a total intravenous anesthetic in dogs. The study found that ketofol induced a biphasic EEG pattern. Subanesthetic doses during induction and recovery led to high beta EEG activity, reflecting increased brain activity. During maintenance with anesthetic doses, the EEG shifted to low beta activity, merging ketamine and propofol EEG characteristics. Ketofol CRI provided robust antinociception activity and maintained a stable heart rate and blood pressure. Subanesthetic ketofol doses caused behavioral excitement, including nystagmus, tongue flicking, salivation and muscle activity. However, these effects were temporary and not a major concern. Such excitement was absent during high-dose general anesthesia. In summary, ketofol CRI showed promise as a safe and effective total intravenous anesthetic combination for dogs. It featured distinct biphasic EEG patterns, stable cardiovascular function and potent antinociception.

**Abstract:**

The effects of concurrent ketamine and propofol (ketofol) constant rate infusion (CRI) were examined in six dogs. The K:P ratio was 1:2, with an initial CRI of 0.25/0.5 mg/kg/min over ten minutes, followed by a 0.5 mg/kg ketamine bolus for induction. During induction, a comprehensive EEG frequency spectrum from delta to gamma was observed, accompanied by subanesthetic-dose ketofol-induced behavioral excitation, including nystagmus, tongue flicking, salivation and active muscle activity. The dogs were maintained on three 15 min decremental doses of ketofol CRI (0.8/1.6, 0.4/0.8 and 0.2/0.4 mg/kg/min). This phase featured a significant decrease in the Patient State Index, electromyographic activity and a shift to low beta waves (SEF95: 13–18 Hz). Additionally, profound antinociception to electric stimulation and a stable heart rate and blood pressure (MBP 81.5–110 mmHg) were observed, as well as a merging of ketamine and propofol EEG characteristics during maintenance. In the recovery phase, a return to beta and gamma EEG patterns and excitement behavior occurred, accompanied by a significant reduction in antinociception, highlighting features of low doses of ketofol. This study reveals biphasic EEG dynamic changes, associated behaviors and robust antinociception and cardiovascular function, suggesting the utility of ketofol as a total intravenous anesthetic combination in dogs.

## 1. Introduction

Propofol is a potent hypnotic agent that enhances GABAa (γ-Aminobutyric acid) receptors within the central nervous system. It offers rapid anesthesia induction and swift recovery whether administered through single injection [1,2] or continuous infusion [2]. However, its use may lead to side effects such as hypotension, bradycardia and respiratory depression, including episodes of apnea and hypoventilation [3,4]. It is important to note that propofol’s analgesic properties are limited [5].

Ketamine, derived from phencyclidine, functions as a non-competitive NMDA (N-methyl D-aspartate) receptor antagonist. Its administration stimulates the sympathetic central nervous system and slows the reuptake of norepinephrine in presynaptic nerves, resulting in increased heart rate and blood pressure [6]. Ketamine can cause respiratory depression, depending on the dose and combination used. Furthermore, it possesses potent analgesic properties even at sub-anesthetic doses, and the duration of its analgesic effect can extend beyond its anesthetic effect. However, the use of ketamine can be associated with side effects such as muscle rigidity, dysphoria and post-operative vomiting [7,8].

The combination of ketamine and propofol, known as “ketofol”, finds widespread use in anesthesia induction and total intravenous anesthesia within human emergency medicine for procedural sedation and analgesia [8,9,10]. This dual-agent approach offers numerous advantages over the individual administration of propofol or ketamine. By incorporating ketamine, the required propofol dosage is reduced. Additionally, ketamine’s stimulatory effect on the autonomic nervous system leads to enhanced cardiovascular stability, a lowered occurrence of apnea or respiratory depression and improved analgesia [9,10]. Furthermore, when administered at lower doses, ketamine minimizes the risk of emergence delirium and postoperative nausea during the recovery period [8,9,10].

In veterinary medicine, researchers have explored the application of ketofol in various animals, including rabbits [11,12], cats [13,14], horses [15] and dogs [16,17,18,19,20,21,22,23]. In dogs, this combination elicits sympathetic stimulation of the cardiovascular system, resulting in an increased heart rate and blood pressure when compared to the use of propofol alone [16,17,18,20,21,22,23]. A study by Henao-Guerrero et al. [21] demonstrated that induction with ketofol led to an augmentation in cardiac output, oxygen delivery and heart rate while maintaining mean arterial pressure when compared to pre-induction values. Unlike humans, dogs show a higher risk of respiratory depression when ketofol is used rather than propofol alone [16,17,19,21,23].

Electroencephalography is now evolving as a standard technique in veterinary research [24,25]. It is widely employed to understand brain activity, evaluate anesthesia depth, investigate neurological disorders and assess the brain effects of pharmacologic agents. With continuous advancements in EEG technology, its applications continue to expand, making it indispensable in both clinical practice and research studies involving dogs [26].

In humans, the electroencephalographic pattern resulting from the combination of propofol and ketamine has been studied. This combination leads to a shift in EEG power, transitioning from the characteristic alpha (10–13 Hz) waves of propofol anesthesia to beta (13–18 Hz) activity when ketamine is introduced [27,28]. Although studies have examined the hemodynamic effects of ketofol, there is currently no available information on the EEG patterns induced by ketofol in dogs to the authors’ knowledge.

The objectives of this study were to evaluate the effects of concurrent ketamine and propofol administration on EEG, antinociception, behavior and select cardiovascular parameters in healthy dogs. We hypothesized that this intravenous anesthesia with ketamine–propofol would yield an intermediate EEG pattern between that of ketamine and propofol when administered individually. Additionally, we expected that the ketofol combination would maintain heart rate and blood pressure while delivering antinociceptive effects.

## 2. Materials and Methods

### 2.1. Study Design

This prospective observational study involved six healthy dogs in 7 distinct phases:

- Phase 0: The study began with six healthy awake dogs.
- Phase 1: Anesthesia induction commenced using ketofol, a combination of ketamine and propofol, administered for 10 min, followed by a bolus of ketamine.
- Phases 2–4: Anesthesia maintenance was carried out using ketofol for 45 min, with this period divided into three 15 min segments. Each segment involved the administration of ketofol at different doses, categorized as high, moderate and low.
- Phase 5: Following the anesthesia maintenance phases, CRI was terminated. Subsequently, the dogs underwent extubation.
- Phase 6: The dogs were monitored until they reached sternal recumbency after extubation.

Throughout each of these phases, the study continuously recorded EEG, EMG and ECG data. Additionally, cardiovascular parameters and antinociceptive testing was conducted at three-minute intervals.

### 2.2. Animals

This study protocol and animal use received approval from the Purdue University Animal Care and Use Committee. Six healthy male intact research beagle dogs, aged 16 months and weighing between 10 and 13 kg, were employed. Prior to the experiment, comprehensive blood work, urinalysis and physical examinations were conducted, all of which yielded results within normal ranges. On the day of anesthesia, the dogs fasted for 8 h while maintaining unrestricted access to water. Additionally, catheters were inserted into the cephalic veins of the dogs.

### 2.3. Drug Administration

The ketofol mixture was prepared by combining 1 mL of ketamine (Zetamine^®^ 100 mg/mL) with a 20 mL vial of propofol (Diprivan^®^ 10 mg/mL), resulting in a final concentration ratio of ketofol (ketamine: propofol) of 4.7:9.5 mg/mL. Continuous rate infusions were administered using a syringe pump, measured in mg per kg per minute.

Prior to induction, baseline values for non-invasive systolic, diastolic and mean blood pressure, heart (pulse) rate, electrocardiogram and processed EEG indices were recorded. After the data were collected while the dogs were awake (Phase 0), they were anesthetized with ketofol CRI at a dosage of 0.25:0.5 mg/kg/min for 10 min, followed by a bolus of ketamine (0.5 mg/kg with a 3-s time frame, Phase 1). They were then intubated and ventilated to maintain end-tidal CO_2_ between 35 and 45 mmHg. EEG data were collected to monitor changes in brain states. 

Anesthesia was maintained for 45 min with decreasing doses of ketofol CRI, starting at 0.8:1.6 mg/kg/minute, decreasing to 0.4:0.8 mg/kg/minute and finally ending in 0.2:0.4 mg/kg/minute in 15 min intervals (Phase 2–4). After the final maintenance dose, CRI was stopped. Then, the dogs were allowed to recover via extubation (Phase 5), and they recovered to sternal recumbency (Phase 6). Continuous monitoring of EEG and cardiovascular parameters (every 3 min) was performed until dogs reached a sternal recumbent position to observe transitions in brain states while regaining consciousness. Throughout the study, the dogs received balanced electrolyte fluid (Plasmalyte^®^) at a rate of 5 mL/kg/h. Data analysis was segmented into 7 distinct phases for statistical evaluation.

### 2.4. EEG and EMG Instrumentation, Measurement and Analysis

The EEG electrode arrangements are detailed in prior works [26,29]. Briefly, the placement of these electrodes followed established guidelines, mirroring the human EEG 10–20 system. The R1 Sedline^®^ electrode corresponds to the Fp2 position in the human 10–20 EEG system. The R2 electrode is positioned between F4 and F8. The L1 electrode is located at Fp1, and the L2 electrode is positioned between F3 and F7. The ground (CB) electrode is placed along the mid-sagittal central line, and the reference (CT) electrodes are cranially situated along the mid-sagittal line. 

The EEG data were continuously and automatically recorded and stored in the .CSV format using Masimo^®^ TraceTM software (V3000). Subsequently, these .CSV files underwent conversion into Excel data files, containing high-resolution processed EEG indices that were recorded at 2 s intervals. These indices included the Patient State Index (PSI), which ranged from 0 (indicating total cortical silence) to 100 (representing complete wakefulness). The Burst Suppression Ratio (SR%) was calculated as the percentage of epochs within the previous 63 s with EEG silent durations longer than 0.5 s and EEG voltages within the range of +5 to −5 μV during this period. Electromyography activity (EMG%), 95% Spectral Edge Frequency (SEF95) for both the left and right hemispheres, being the frequency below which 95% of the total EEG power is located, and artifact activity (ART%) were also part of the recorded indices. Additionally, for the visual examination of raw EEG recordings, an .edf file was downloaded from the monitor.

The Sedline EEG monitor tracks EMG activity in the facial and forehead muscles through electrodes placed on the dog’s forehead. These electrodes measure the electrical activity of these muscles, including those involved in actions such as grimacing, jaw clenching, eye blinking, nystagmus, chewing, swallowing and vocalizing. The Sedline EEG monitor then utilizes these EMG data as a muscle relaxation component contributing to the calculation of a PSI value, which reflects the overall sedative/anesthetic activity.

Prior to each study, thorough inspections and validations of these modified electrodes were conducted using the Sedline Root^®^ monitor. The acceptance criteria encompassed impedance values within the range of 0.0 to 65.1 kilo-ohms, visually indicated by a green icon displayed at the top of the monitor screen for each electrode. In cases of non-compliance with these criteria, a red indicator alerted the need for immediate electrode replacement until acceptable impedance levels were attained.

By utilizing subdermal needle electrode modification, there was no need for hair clipping or skin degreasing, streamlining the electrode placement process in the dogs. Maintaining the needles in place proved to be straightforward.

Throughout the anesthesia duration, the dogs were monitored using a traditional multiparameter monitor (Digicare LW9XVet, Lifewindow, Digicare Biomedical, Boynton FL, USA). Key parameters including electrocardiogram, oscillometric blood pressure, hemoglobin saturation of oxygen with pulse oximetry (SpO_2_), respiratory rate, end-tidal CO_2_ and rectal temperature were recorded at 3 min intervals during the experiment. For cardiovascular assessment, cardiac arrhythmias were monitored using a lead II electrocardiogram. Oscillometric measurements of systolic, diastolic and mean arterial blood pressure were measured using a blood pressure cuff placed on one of the hindlimbs, specifically in the metatarsal bone area, with the cuff width adjusted to approximately 40% of the limb’s metatarsal circumference. SpO_2_ was measured using a lingual probe placed on the dog’s tongue. After tracheal intubation, the dog was connected to sidestream capnography to measure end-tidal CO_2_ levels and respiratory rate. The dogs were mechanically ventilated using a pressure-controlled ventilator at a rate of 8–12 breaths per minute, a tidal volume of 15 mL/kg and a peak inspiratory pressure of 15–20 cm of water. These ventilator parameters were fine-tuned to maintain end-tidal CO_2_ levels between 35 and 45 mmHg and an SpO_2_ of 98–100%. All these parameters were taken before the antinociception test. The dog’s body temperature was maintained between 99 and 101 F using towels and a heating pad, and 100% oxygen was provided through the mechanical ventilator via an anesthetic rebreathing circuit attached to an anesthetic machine.

### 2.5. Antinociception Tests

To gauge antinociception levels during both the anesthesia maintenance and recovery phases, we employed electrical stimulation. This method has been shown to be effective and was previously used to assess antinociception during anesthesia [26,30]. Specifically, we inserted two 25-gauge needles into the subcutaneous tissue over the lateral aspect of the tibia, maintaining a 5 cm separation between them. These electrodes were then connected to a nerve stimulator set to tetanus mode, which administered a square wave pulse stimulus lasting 0.22 milliseconds at 400 V. The stimulator allowed us to adjust the intensity settings, ranging from 0 to 100 pulses per second (Hz). In this scale, 1 denotes the lowest intensity setting (0 Hz), whereas 9 indicates the highest intensity setting (100 Hz). We administered stimulation every three minutes, synchronized immediately after collecting physiological data. Each stimulation lasted 2 s, starting with the lowest intensity setting. A positive response was characterized by a heart rate increase exceeding 20 beats per minute or observable purposeful movements, encompassing limb withdrawal, head elevation or neck motion, tail or nasal twitching, swallowing or active blinking. We continued to increment the stimulation intensity until we observed a positive response or reached the predetermined maximum stimulation level (900 Hz).

### 2.6. Behavioral Assessment

During the induction phase, any signs of excitation associated with ketamine–propofol such as tongue flicking, paddling or nystagmus were carefully observed and documented for each dog. The dog’s auditory response was tested throughout the experiment by making two clicks near each ear [30]. If the dog turned its head toward the source of the noise, it was considered to have a positive auditory response [30]. Additionally, a subjective depth score of anesthesia (Appendix A), previously utilized in our research [26], was employed to assess the depth of anesthesia every 3 min.

Upon reaching the recovery phase, and as the dogs resumed spontaneous breathing, they were removed from the ventilator and allowed to breathe spontaneously. Extubation took place when clinical signs of endotracheal tube intolerance, such as swallowing, coughing or gagging, were detected, and the time of extubation was recorded. Any other unusual behavioral signs during the recovery phase, including active blinking, nystagmus, tongue flicking, vocalization or excessive muscle activities, were observed and documented.

### 2.7. Statistics

The 2 s EEG data were utilized for statistical analysis, resulting in over 20,000 data points for certain variables. In the case of SEF95 data, calculations were performed separately for the left and right hemispheres, and hemispheric coherence was compared using paired *t*-tests. 

For the construction of the DSA, we used the raw EEG as recorded from the SEDLine monitor. Because of a known technical issue, we re-sampled all recorded EEG to a sampling rate of 89 Hz [31]. We constructed the DSA using the MATLAB pwelch function (Matlab R2019a, The MathWorks, Natick, MA, USA) with a frequency resolution of 0.35 Hz. The DSA was calculated from 5 s EEG with a 1 s shift. 

To analyze the PSI, SR, SEF95, EMG and ART data for each dog, the data were grouped by phase. Mean values for each dog were then compared across phases using linear mixed model analyses with repeated measurements. Statistical significance was determined with a *p*-value < 0.05. If phase data significantly differed overall for a specific variable, pairwise comparisons were conducted using Bonferroni correction for multiple comparisons. All data were presented with the mean ± SD for both hemodynamic and EEG indices.

## 3. Results

The EEG indices and hemodynamic data for each experimental phase are presented in Table 1 and Table 2, respectively. Biphasic EEG patterns were observed during the induction and maintenance phases, returning to a pattern resembling the induction phase during recovery.

### 3.1. EEG and Behavior Changes during Induction 

In the awake state, key EEG characteristics include a high PSI value (>90), signaling a state of alertness, and prominent muscle activity, as evidenced by elevated EMG readings. Notably, there was an absence of slow-wave patterns, and the EEG displayed a predominance of high beta and gamma wavelength activity (Table 1). All dogs exhibited a reaction to the auditory test.

**Figure 1:**
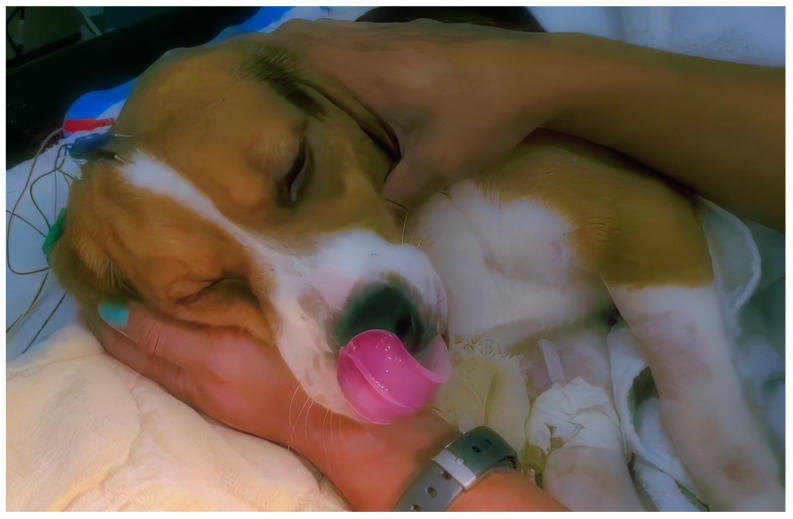
This figure illustrates tongue flicking (nose licking) with salivation behavior in dogs during ketamine–propofol CRI induction and recovery phases.

During the induction phase, three out of the six dogs displayed signs of ketamine–propofol-induced excitement, including mild head shaking, active eye blinking, nystagmus, muscle rigidity and tongue flicking (or nose licking) (Figure 1) with salivation. These signs persisted until a ketamine dose of 0.5 mg/kg was administered. After administering the ketamine bolus, dogs were assessed for tracheal intubation readiness, indicated by minimal resistance to tongue withdrawal, a relaxed jaw tone and the absence of coughing and gagging during tracheal placement. Additionally, EEG readings confirmed the dogs’ sufficient anesthetic depth, aligning with clinical signs. Tracheal intubation was consistently performed by investigator CM and was achieved swiftly following the ketamine bolus. In the early induction phase, auditory responses became inconsistent, and all dogs lost their auditory response following endotracheal intubation.

**Figure 2:**
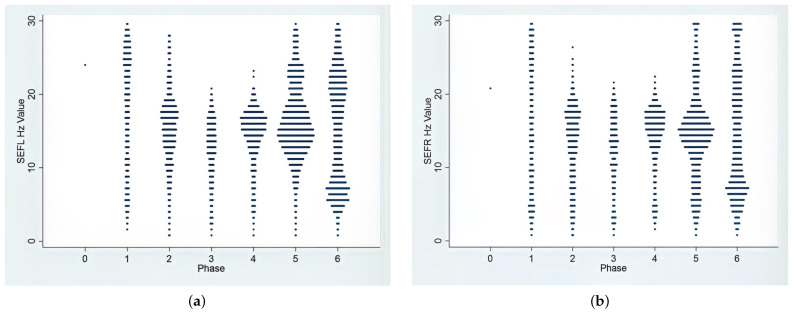
Frequency distributions of SEF95% (the frequency at which 95% of the total EEG power is located) in the left (**a**) and right (**b**) hemispheres during the 7 phases of anesthesia from induction to recovery. During the anesthetic procedure, the dominant frequency range underwent a biphasic shift. Initially, during the induction phase, it showed high beta and gamma waves ranging from 20 to 35 Hz. Subsequently, in the maintenance phases (Phases 2–4), there was a transition to low beta waves with frequencies between 13 and 18 Hz. Finally, during the recovery phases, there was a return to beta and gamma waves. In the awake phase (Phase 0), owing to substantial artifacts and muscle activities, the monitor recorded minimal values for SEF95% data points.

The EEG recordings during induction revealed a broad range of frequencies, spanning from slow delta to theta and extending to gamma waves. Figure 2 provides a visual representation of the SEF95 values corresponding to these waveforms. Also, heart rate significantly increased from the baseline, with minimal changes in arterial blood pressures, as detailed in Table 2. During the induction phase, a light plane of anesthesia was observed in the dogs, as indicated by their subjective depth score in Table 2. Due to this light plane of anesthesia and the presence of auditory responses, nociception testing was not conducted at this stage. As the induction phase progressed, a gradual decrease in PSI values was noted, indicating a reduction in wakefulness. Additionally, a slight increase in slow-wave patterns and the emergence of low beta wavelength frequencies in the raw EEG were observed. It is worth mentioning that the auditory response disappeared during this phase.

### 3.2. EEG Indices, Hemodynamic and Antinociceptive Data during CRI Maintenance

**Figure 3:**
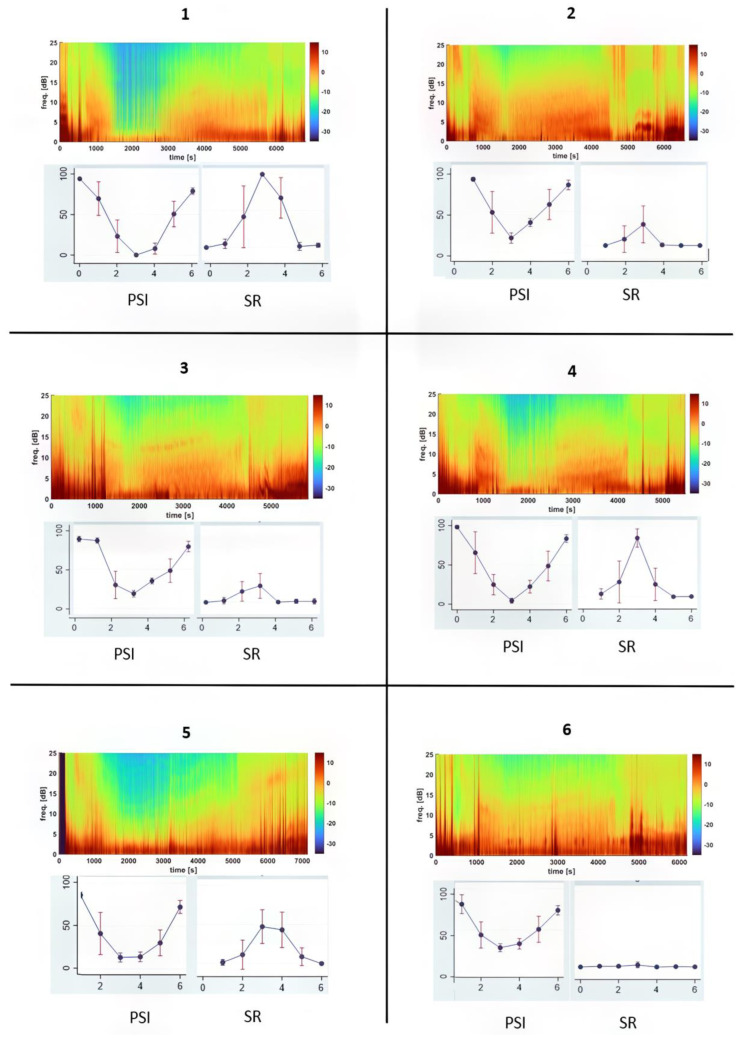
This figure illustrates the brain state changes observed in six dogs undergoing ketofol CRI anesthesia. It provides an overview of each dog’s response to anesthesia by displaying the median Density Spectral Array (DSA), Patient State Index (PSI) and Suppression Ratio (SR). The DSA graphs reveal distinct EEG patterns that evolve over time, each represented by a unique color spectrum corresponding to an individual dog’s response. Notably, there is a significant contrast between Dog #1 and Dog #6. Dog #1’s DSA shows a pillar pattern of burst suppression around 2000 s, whereas Dog #6 shows a niche continuous EEG pattern. On the PSI graph, Dog #1 consistently displays considerably lower PSI values compared to Dog #6 during the CRI maintenance phases, indicating a deeper level of brain depression. Additionally, the SR values, expressed as percentages, exhibit marked differences between these two dogs, with Dog #1 consistently showing higher values than those of Dog #6. This discrepancy suggests a more profound suppression of brain activity in Dog #1 during Phases 2–4, highlighting distinct sensitivities in their respective responses to ketofol anesthesia.

Ketofol CRI gradually deepened anesthesia to its deepest level in Phase 3, as shown by a lower PSI, higher SR, lower blood pressure and stronger antinociception and muscle relaxation (Table 1 and Table 2 and Figure 3).

In Phase 4, characterized by the lowest dose of ketofol CRI, the plane of anesthesia began to lighten. This was evident through an increase in PSI, a rise in blood pressure and heart rate, a reduction in the SR value and a decreased tolerance to electrical stimulation. The raw EEG waves during the CRI maintenance phases were mainly alpha and low beta waves (13–20 Hz), as shown in Table 1 and Figure 2.

### 3.3. EEG and Hemodynamic Values and Behavior Signs during Recovery

Following the termination of CRI, the dogs transitioned from unconsciousness to consciousness, manifesting this shift by exhibiting intolerance to the endotracheal tube, accompanied by coughing and gagging reflexes. The mean duration from the conclusion of CRI to extubation was 24 min, with a range of 17 to 42 min. Following extubation, it took an average of 23 min for dogs to assume sternal recumbency, with a range between 16 and 31 min. The PSI value increased to 47.4 ± 18.7 at extubation and peaked at 78.1 ± 7.4 when they resumed sternal recumbency (Table 1 and Figure 3). Simultaneously, the SR decreased significantly, nearly reaching zero. Muscle activity began to return and gain strength during this phase.

After extubation, three dogs exhibited mild head shaking, tongue flicking and muscle activity behavior. These behaviors were self-limiting and did not necessitate any intervention. Notably, during this phase, the PSI increased to levels resembling those observed during the induction phase. Furthermore, both heart rate and blood pressure returned to and, in some cases, exceeded the awake baseline values.

An interesting observation during the recovery phases was the shift in EEG waveforms. These shifts encompassed both an upward trend toward gamma waves and a downward trend toward delta and theta waves in both directions, as visually represented in Figure 2.

### 3.4. Individual Dog Sensitivity and Responses to Ketamine–Propofol CRI Anesthesia Based on EEG Indices

**Figure 4:**
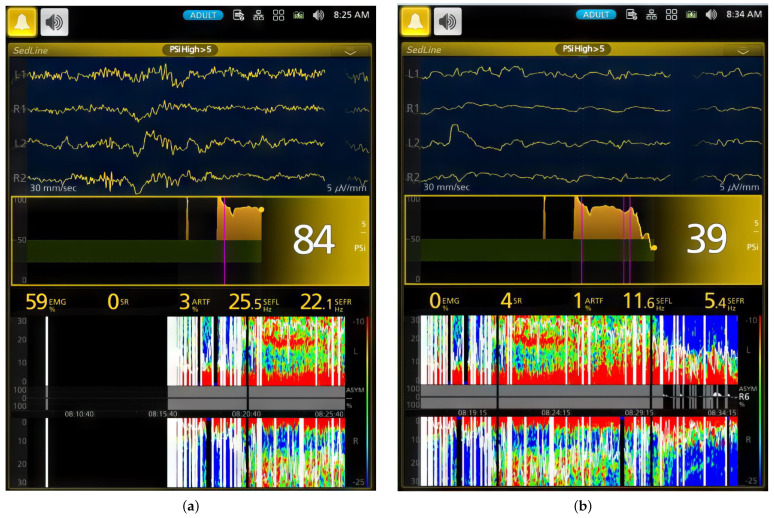
This figure illustrates two key moments during the ketamine–propofol CRI induction (**a**) and early maintenance phase (**b**) of a study dog. The first EEG screenshot shows a dog that is close to wakefulness, with a high PSI of 84, high EMG activity of 59% and high beta wave activity of 25.5 Hz (left hemisphere) and 22.1 Hz (right hemisphere). The second screenshot shows the same dog under anesthesia, with a low PSI of 39, EMG activity of 0% and low beta wave activity of 11.6 Hz (left hemisphere) and 5.4 Hz (right hemisphere). (**b**) In this phase, the dog’s PSI decreases from 84 to 39 after a 0.5 mg/kg ketamine bolus, marking a shift to CRI maintenance (second pink line; first line is CRI start, and third line is intubation completion). The DSA shows a transition from high-frequency gamma and beta waves to dominant low-frequency alpha and delta waves with a zipper opening pattern. SEF95 values are 11.6 Hz and 5.4 Hz. The PSI trend indicates deeper anesthesia over time, with a Burst Suppression Ratio of 4.

**Figure 5:**
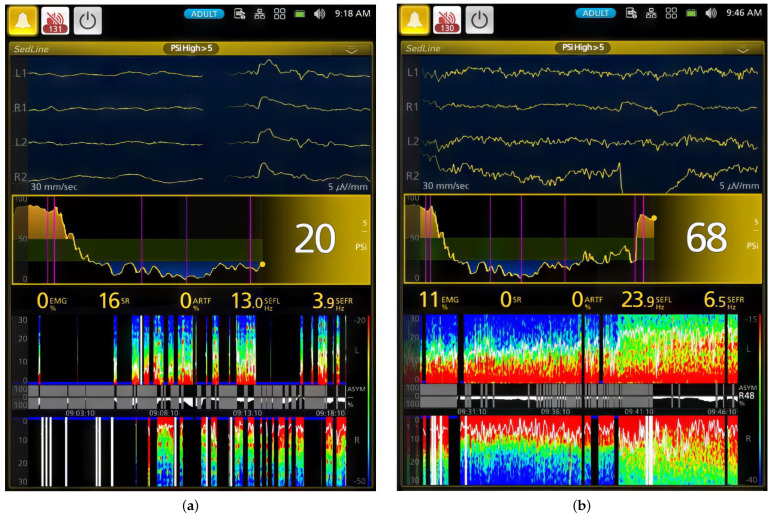
This figure presents two key snapshots of a dog during ketamine–propofol CRI maintenance (**a**) and recovery phases (**b**). The snapshots show distinct stages of anesthesia. (**a**) In the first snapshot, the PSI is 20, indicating deep general anesthesia and unconsciousness. EMG activity is at 0%, signifying muscle relaxation. SEF95 values are 13.0 Hz (left hemisphere) and 3.9 Hz (right hemisphere), indicating alpha and delta wave activity. The DSA shows a burst suppression pattern with low-frequency delta and theta waves. The PSI trend declines from consciousness to deep anesthesia, marked by the first pink vertical line. The Burst Suppression Ratio is 16%. (**b**) In the second snapshot, the PSI returns to 68 after CRI administration termination, marked by the 6th vertical pink line. The DSA shows a shift from delta and theta waves to high-frequency beta and gamma waves. SEF95 values are 23.9 Hz and 6.5 Hz, signifying high beta and theta activities. The PSI trend reflects the transition from unconsciousness to consciousness during ketamine–propofol CRI anesthesia. The Burst Suppression Ratio is 0 with 11% muscle activity during early recovery.

In Figure 3, the median DSA (Density Spectral Array) for each dog is presented to visualize individual responses to ketofol CRI throughout anesthesia. This figure effectively demonstrates changes in EEG frequency and power over time, with a specific focus on PSI and SR values for each dog. Dogs had different PSI and SR values, especially Dog #1 and Dog #6. Dog #1 had a much lower (*p* < 0.001) PSI value and a much higher %SR than those of Dog #6 during the maintenance phases (Phases 2–4) in response to ketofol. Their DSA power graphs were completely different (Figure 3). Figure 4 and Figure 5 display real-time EEG-based brain state changes in a study dog during various phases of ketofol anesthesia.

## 4. Discussion

In this study, we investigated the EEG patterns, cardiovascular responses, antinociceptive effects and behavioral alterations in dogs under ketamine–propofol constant rate infusion anesthesia. Our findings reveal significant dynamic changes in DSA and EEG indices (PSI, SR and EMG) across various anesthesia phases. These alterations corresponded with changes in antinociceptive efficacy, cardiovascular parameters and subjective evaluations of anesthetic depth.

During induction, dogs underwent 10 min of CRI of ketofol without a loading dose or premedication. Although this approach allowed us to observe brainwave and behavior changes during this period, it has certain clinical disadvantages. For instance, some dogs may experience paradoxical excitation, as observed in a previous propofol study [26]. Pain upon injection may also occur with propofol and ketamine. However, preplacing an intravenous catheter can likely help mitigate or alleviate the irritation caused by these drug-induced pains. During this induction phase, EEG patterns displayed a wide range of frequencies from delta to gamma waves. This diverse spectrum implies the effects of ketamine and propofol on target receptors, specifically NMDA and GABAa receptors, as supported by prior research [32,33,34].

Three dogs displayed behavioral excitement during induction, including mild head shaking, active eye blinking, nystagmus, muscle rigidity and tongue flicking. These signs persisted despite propofol coadministration. Bolus administration of 0.5 mg/kg of ketamine was necessary to deepen the anesthesia for endotracheal intubation, as we learned from our pilot study. The excitement behaviors subsided after the bolus ketamine induction, and successful endotracheal intubation was achieved in all dogs.

The behavioral excitement observed during the induction phase can be attributed to elevated beta and gamma wave activity, signifying increased brain activity. Ketamine, at low to moderate doses, primarily blocks NMDA receptors in the cortex and subcortical structures, leading to heightened thalamocortical pathway activity and reduced hippocampal activity. Consequently, this manifests as clinical signs of excitement and dissociation. However, the administration of a ketamine bolus raised the concentration to a higher threshold. At high doses, ketamine also blocks NMDA receptors in the thalamus and brainstem, resulting in decreased thalamocortical pathway activity, ultimately leading to general anesthesia [27,32,33,34,35,36,37,38,39].

This explanation gains further support from the observed cessation of these behaviors after the ketamine bolus administration. Additionally, the EEG wave patterns underwent a significant shift, transitioning from gamma and high beta waves to low beta and alpha waves. Simultaneously, there was a decrease in PSI coupled with an increase in SR. These combined changes indicate that ketamine reached the threshold required to induce general anesthesia. In conjunction with propofol, it achieved a state of general anesthesia in the brain.

While maintaining CRI, initial signs of behavioral excitation diminished in alignment with the biphasic EEG pattern shift from the induction transition to the maintenance phase. However, these signs reappeared and intensified during the subsequent recovery phase after discontinuing CRI. Simultaneously, EEG recordings consistently displayed heightened high-frequency beta and gamma waves during these phases, signifying a resurgence of increased brain activity as ketamine–propofol concentrations decreased. This suggests that the pronounced behavioral excitation observed during the recovery phase was likely a result of the lingering effects of ketamine, often referred to as the “ketamine hangover” [40,41] effect, coupled with the decrease in the anesthetic effect of propofol. EEG changes during this phase further confirm this interpretation, showing an increase in brain activity.

Furthermore, EEG patterns serve as a valuable tool to differentiate between low to moderate doses and high doses of ketamine with propofol. At low to moderate doses, the EEG reveals heightened high beta and gamma activity along with reduced alpha activity. In contrast, at high doses during the maintenance phases, the EEG undergoes a shift from high beta to low beta activity, accompanied by increased alpha and delta activity, demonstrating a biphasic dynamic shift. These EEG findings closely parallel those reported in studies involving ketamine or ketamine in combination with propofol in humans or non-human primates [27,32,33,34,35,36,37,38,39].

General anesthesia was effectively maintained throughout Phases 2–4 of the CRI, reaching its deepest plane during Phase 3, as indicated by the EEG indices. General anesthesia was deepest in Phase 3 because the anesthetic drugs had more time to accumulate in the body, even though the CRI drug concentration was lower than that in Phase 2. This finding underscores the significance of both concentration and duration in determining anesthesia depth, with time playing a role in achieving deeper anesthetic levels.

Cardiovascular functions remained stable, characterized by a well-maintained heart rate and blood pressure, with the lowest mean arterial blood pressure recorded at 81.5 ± 14.7 mmHg during this deepest stage of anesthesia. Importantly, none of the dogs exhibited hypotension or bradycardia.

Although it is challenging to make a direct comparison with our previous study [26] involving CRI with propofol alone in dogs, it is worth mentioning that, in that study, blood pressure levels were lower, and hypotension occurred during the propofol CRI maintenance stages. In the current study, the benefits observed can be attributed to the combined use of ketamine alongside lower doses of propofol. These dual beneficial effects likely played a significant role in maintaining sympathetic stimulation. This, in turn, had a notable impact on upholding chronotropic and inotropic effects in the dogs under investigation. This combination appears to have contributed positively to the overall outcomes of the study.

The well-maintained anesthesia was further reflected in the robust antinociceptive activity. During the maintenance phases, electric stimulation elicited values near the maximum (ranging from 800 to 875 vs. a maximum of 900). These values decreased to 629.1 ± 231.8 Hz upon termination of CRI but continued to linger for approximately 24 min before diminishing entirely. The decline in antinociceptive activity can be attributed to a combination of a decreasing ketamine plasma concentration and the absence of propofol’s anesthetic effect. This is evident from the notable increase in PSI values and the fading of ketamine’s EEG signature, characterized by low beta waves, during Phase 5.

One valuable aspect of employing EEG for monitoring anesthesia depth is its ability to provide real-time differentiation of individual responses to anesthetics [32]. When applied in the operating room, this enables the titration of anesthetic depth according to individual responses across various anesthesia phases [32]. This functionality of EEG is effectively demonstrated in the current ketofol CRI study, where we can distinctly identify each individual dog’s PSI, SR and DSA (Figure 3, Figure 4 and Figure 5). Moreover, it allows for clear and continuous tracking of anesthesia depth throughout each phase of the procedure.

Throughout the study, the dynamic response of the EEG to varying concentrations of ketofol was observed. Notably, a biphasic shift in EEG dynamics occurred when ketofol administration was terminated during Phases 5 and 6. These alterations in EEG dynamics are clearly depicted in Figure 2 (SEF) and Figure 3 (DSA) and signify a decrease in both ketamine and propofol concentrations. These EEG changes are consistent with the “ketamine hangover” mentioned earlier, which was observed in three dogs.

EEG is a fairly reliable tool for monitoring anesthesia depth in both humans [32,33,34] and dogs [26], as evidenced in our prior research. However, it is essential to acknowledge its imperfections, as factors like age, health status and anesthetic drugs can influence its accuracy. Interpreting EEG can be intricate and demands appropriate training. Modern EEG monitors not only provide processed EEG indices but also offer raw EEG and DSA data, as highlighted in our recent study, which enhances EEG’s dependability in dogs [26]. Although EEG is a valuable tool for anesthesia depth monitoring, it should be used alongside other clinical parameters, such as heart rate, blood pressure, nociceptive responses and subjective anesthetic depth scores derived from reflexes and behavioral observations, as executed in our study.

This study had some limitations, including a small sample size, a fixed ketamine–propofol ratio and limited cardiovascular monitoring. This study also used mechanical ventilation, which may have varied throughout anesthesia. Despite these limitations, this study established an EEG pattern for ketofol anesthesia, demonstrating the potential of EEG for monitoring anesthesia depth and individual responses.

The choice of decremental dosing for CRI offers the advantage of a faster onset of anesthesia with reduced overall drug consumption. It also allows for rapid tapering off of the “ketamine hangover” as CRI doses are reduced proportionally. Conversely, incremental dosing requires more monitoring to tailor anesthesia to individual needs but is less likely to lead to an overshoot of the desired anesthetic level. In our pilot study, achieving the desired anesthetic level with incremental dosing took a significant amount of time, especially without the use of a bolus loading dose. Therefore, for this study, we opted for a decremental dose CRI approach. Nonetheless, this approach enabled us to observe the biphasic behavioral effects of ketamine–propofol and its corresponding EEG patterns. Understanding drug metabolism and excretion is crucial for drug administration. It affects how long a drug stays in the body and its effectiveness. Decremental doses aim to minimize side effects, but in this study using ketamine–propofol CRI, some dogs still showed ketamine “hangover” signs despite achieving anesthetic maintenance. 

## 5. Conclusions

This study presents dynamic changes in EEG, antinociception and behavior linked to ketamine–propofol constant rate infusion anesthesia in dogs. It also underscores the ability to monitor individual dog responses to ketofol anesthesia across the various anesthesia phases. Additionally, the study results suggest that ketamine with propofol CRI is likely a valuable total intravenous anesthetic combination. This combination accomplishes unconsciousness and robust antinociception and does not appear to induce obvious cardiovascular adverse reactions throughout the study.

## Figures and Tables

**Table 1:** Mean EEG index values (±SD) observed in six dogs during coadministration of ketamine and propofol anesthesia. Values with different superscripts differ significantly (*p* < 0.0001). EMG, ART, SEFR and SEFL values do not show significant differences from each other across all phases. Please refer to the text for the abbreviations of the processed EEG indices.

Phase	PSI	SR (%)	EMG (%)	ARTF (%)	SEFR (Hz)	SEFL (Hz)
0	92.7 ± 5.1 ^a^	0.0 ± 0.0 ^a^	52.4 ±30.0	9.8 ± 9.6	20.8 ± 0.0	23.6 ± 0.0
1	79.9 ± 17.1 ^a^	2.4 ± 5.0 ^b^	57.9 ± 28.6	5.4 ± 10.2	15.3 ± 8.4	18.0 ± 7.6
2	35.7 ± 22.5 ^b^	16.6 ± 27.4 ^b^	7.8 ± 14.0	4.7 ± 14.3	13.3 ± 5.0	15.3 ± 4.8
3	14.0 ± 10.4 ^c^	46.7 ± 37.2 ^c^	1.0 ± 1.8	0.1 ± 0.8	12.3 ± 4.8	12.4 ± 4.2
4	25.0 ± 12.7 ^b^	21.7 ± 31.0 ^b^	1.9 ± 3.1	0.4 ± 2.2	14.2 ± 4.1	14.4 ± 3.7
5	47.4 ± 1 8.7 ^b^	2.4 ± 6.6 ^b^	12.4 ± 17.3	0.6 ± 3.0	14.4 ± 5.7	16.4 ± 4.9
6	78.1 ± 7.4 ^a^	0.6 ± 2.1 ^a^	32.5 ± 22.8	10.4 ± 17.1	14.0 ± 7.8	15.1 ± 7.4

**Table 2:** Mean values (±SD) for heart rate (HR), systolic blood pressure (SBP), mean arterial blood pressure (MBP), diastolic blood pressure (DBP), subjective anesthetic depth score and tolerance to electric stimulation across seven phases of ketamine–propofol continuous rate infusion anesthesia in six dogs. Values with different superscripts differ significantly (*p* < 0.0001).

Phase	HR (bpm)	SBP (mmHg)	MBP (mmHg)	DBP (mmHg) ^c^	Depth Score ^d^	Electrical Stimulation (Hz) ^e^
0	108.9 ± 21.0	165.5 ± 22.4	114.5 ± 15.9	96.7 ± 15.1	5.0 ± 0.0 ^a^	NA
1	135.7 ± 35.1	164.1 ± 17.9	114.4 ± 22.6	97.3 ± 25.4	4.4 ± 0.6 ^a^	NA
2	142.9 ± 21.3	149.3 ± 28.6	105.1 ± 25.4 ^a^	89 ± 26.8 ^a^	2.7 ± 0.7 ^b^	845.4 ± 126.2 ^a^
3	131.2 ± 12.6	123.6 ± 21.5 ^a^	81.5 ± 14.7 ^a^	67.4 ± 14.6 ^a^	2.2 ± 0.4 ^b^	875 ± 56.8 ^a^
4	146.1 ± 15.2	144.4 ± 25.0	110.3 ± 23.3	93.9 ± 21.0 ^a^	2.4 ± 0.5 ^b^	803.0 ± 170.4 ^a^
5	140.9 ± 22.3	171.7 ± 17.5 ^b^	137.7 ± 13.6 ^b^	118.8 ± 12.8 ^b^	3.2 ± 0.6 ^c^	629.1 ± 231.8 ^a^
6	162.0 ± 25.1	187.2 ± 32.9 ^b^	147.2 ± 29.2 ^b^	133.3 ± 26.9 ^c^	4.9 ± 0.2 ^a^	150 ± 0.0 ^b^

## Data Availability

The data are contained within the article.

## Appendix A

**Table A1:** Subjective anesthetic depth score in dogs [26]. The palpebral reflex was assessed with a cotton swab, and the corneal reflex was assessed with sterile saline drops on the corneal surface via syringe.

Anesthetic Plane	Depth Score	Pupil Size	Globe Position	Eye Reflex (P: Palpebral; C: Corneal)	Heart Rate, Blood Pressure, Breathing	Third Eyelid Position	Jaw Tone, Body/Limb Movement or Swallowing
Very light	5	Large or small, moving	Central	P: active C: Active	Increased, spontaneous, breathing attempts	Retracted	Strong
Medium-light	4	Large or small	Central or ventromedial	P: active or mildly depressed C: depressed	Increased, spontaneous breathing attempts	Retracted or partially prolapsed	Medium
Medium	3	Small or medium	Ventromedial	P: depressed C: partially depressed	Normal	Completely prolapsed	None or barely
Medium-deep	2	Medium	Central	P: depressed or markedly depressed C: depressed	Normal or depressed	Completely or partially prolapsed	None
Very deep	1	Large	Central	P: absent C: markedly depressed or absent	Depressed	Partially depressed	None

## References

[1] Glen J.B. (1980). Animal studies of the anaesthetic activity of ICI 35 868. Br. J. Anaesth..

[2] Sebel P.S., Lowdon J.D. (1989). Propofol: A new intravenous anesthetic. Anesthesiology.

[3] Marik P.E. (2004). Propofol: Therapeutic Indications and Side-Effects. Curr. Pharm. Des..

[4] Sahinovic M.M., Struys M.M.R.F., Absalom A.R. (2018). Clinical Pharmacokinetics and Pharmacodynamics of Propofol. Clin. Pharmacokinet..

[5] Vasileiou I., Xanthos T., Koudouna E., Perrea D., Klonaris C., Katsargyris A., Papadimitriou L. (2009). Propofol: A review of its non-anaesthetic effects. Eur. J. Pharmacol..

[6] Li L., Vlisides P.E. (2016). Ketamine: 50 Years of Modulating the Mind. Front. Hum. Neurosci..

[7] White P.F., Way W.L., Trevor A.J. (1982). Ketamine—Its Pharmacology and Therapeutic Uses. Anesthesiology.

[8] Andolfatto G., Abu-Laban R.B., Zed P.J., Staniforth S.M., Stackhouse S., Moadebi S., Willman E. (2012). Ketamine-Propofol Combination (Ketofol) Versus Propofol Alone for Emergency Department Procedural Sedation and Analgesia: A Randomized Double-Blind Trial. Ann. Emerg. Med..

[9] Smischney N.J., Beach M.L., Loftus R.W., Dodds T.M., Koff M.D. (2012). Ketamine/propofol admixture (ketofol) is associated with improved hemodynamics as an induction agent: A randomized, controlled trial. J. Trauma Acute Care Surg..

[10] Jalili M., Bahreini M., Doosti-Irani A., Masoomi R., Arbab M., Mirfazaelian H. (2016). Ketamine-propofol combination (ketofol) vs propofol for procedural sedation and analgesia: Systematic review and meta-analysis. Am. J. Emerg. Med..

[11] Gonz A. (2018). Corticoadrenal and Cardiorespiratory Responses to Administration of Propofol Combined with Dexmedetomidine or Ketamine in Rabbits. J. Am. Assoc. Lab. Anim. Sci..

[12] Hashemi S.R., Vesal N. (2023). Ketamine–propofol for total intravenous anaesthesia in rabbits: A comparison of premedication with acepromazine–medetomidine, acepromazine–midazolam or acepromazine–morphine. Vet. Anaesth. Analg..

[13] Boudreau A.E., Bersenas A.M.E., Kerr C.L., Holowaychuk M.K., Johnson R.J. (2012). A comparison of 3 anesthetic protocols for 24 h of mechanical ventilation in cats. J. Vet. Emerg. Crit. Care.

[14] Ravasio G., Gallo M., Beccaglia M., Comazzi S., Gelain M.E., Fonda D., Bronzo V., Zonca A. (2012). Evaluation of a ketamine-propofol drug combination with or without dexmedetomidine for intravenous anesthesia in cats undergoing ovariectomy. J. Am. Vet. Med. Assoc..

[15] Jarrett M.A., Bailey K.M., Messenger K.M., Prange T., Gaines B., Posner L.P. (2018). Recovery of horses from general anesthesia after induction with propofol and ketamine versus midazolam and ketamine. J. Am. Vet. Med. Assoc..

[16] Kennedy M.J., Smith L.J. (2015). A comparison of cardiopulmonary function, recovery quality, and total dosages required for induction and total intravenous anesthesia with propofol versus a propofol-ketamine combination in healthy Beagle dogs. Vet. Anaesth. Analg..

[17] Mair A.R., Pawson P., Courcier E., Flaherty D. (2009). A comparison of the effects of two different doses of ketamine used for co-induction of anaesthesia with a target-controlled infusion of propofol in dogs. Vet. Anaesth. Analg..

[18] Lee M., Kim S., Moon C., Park J., Lee H., Jeong S.M. (2017). Anesthetic Effect of Different Ratio of Ketamine and Propofol in Dogs. J. Vet. Clin..

[19] Lerche P., Reid J., Nolan A.M. (2000). Comparative study of propofol or propofol and ketamine for the induction of anaesthesia in dogs. Vet. Rec..

[20] Martinez-Taboada F., Leece E.A. (2014). Comparison of propofol with ketofol, a propofol-ketamine admixture, for induction of anaesthesia in healthy dogs. Vet. Anaesth. Analg..

[21] Henao-Guerrero N., Riccó C.H. (2014). Comparison of the cardiorespiratory effects of a combination of ketamine and propofol, propofol alone, or a combination of ketamine and diazepam before and after induction of anesthesia in dogs sedated with acepromazine and oxymorphone. Am. J. Vet. Res..

[22] Wamaitha M., Mogoa E., Mande J. (2019). Evaluation of anesthesia produced by ketofol in acepromazine- or medetomidine-sedated dogs. J. Adv. Vet. Anim. Res..

[23] Seliškar A., Nemec A., Roškar T., Butinar J. (2007). Total intravenous anaesthesia with propofol or propofol/ketamine in spontaneously breathing dogs premedicated with medetomidine. Vet. Rec..

[24] Silva A., Antunes L. (2012). Electroencephalogram-based anaesthetic depth monitoring in laboratory animals. Lab. Anim..

[25] Drinkenburg W.H., Ahnaou A., Ruigt G.S. (2015). Pharmaco-EEG Studies in Animals: A History-Based Introduction to Contemporary Translational Applications. Neuropsychobiology.

[26] Murillo C., Weil A.B., Moore G.E., Kreuzer M., Ko J.C. (2023). Electroencephalographic and Cardiovascular Changes Associated with Propofol Constant Rate of Infusion Anesthesia in Young Healthy Dogs. Animals.

[27] Hayashi K., Tsuda N., Sawa T., Hagihira S. (2007). Ketamine increases the frequency of electroencephalographic bicoherence peak on the α spindle area induced with propofol. Br. J. Anaesth..

[28] Sakai T., Singh H., Mi W.D., Kudo T., Matsuki A. (1999). The effect of ketamine on clinical endpoints of hypnosis and EEG variables during propofol infusion. Acta Anaesthesiol. Scand..

[29] Murillo C., Weng H.-Y., Weil A.B., Kreuzer M., Ko J.C. (2022). Perioperative Brain Function Monitoring with Electroencephalography in Horses Anesthetized with Multimodal Balanced Anesthetic Protocol Subjected to Surgeries. Animals.

[30] Krimins R.A., Ko J.C., Weil A.B., Payton M.E. (2012). Evaluation of anesthetic, analgesic, and cardiorespiratory effects in dogs after intramuscular administration of dexmedetomidine–butorphanol–tiletamine-zolazepam or dexmedetomidine-tramadol-ketamine drug combinations. Am. J. Vet. Res..

[31] Von Dincklage F., Jurth C., Schneider G., Garcia P.S., Kreuzer M. (2021). Technical considerations when using the EEG export of the SEDLine Root device. J. Clin. Monit. Comput..

[32] Purdon P.L., Sampson A., Pavone K.J., Brown E.N. (2015). Clinical Electroencephalography for Anesthesiologists. Anesthesiology.

[33] Purdon P.L., Pierce E.T., Mukamel E.A., Prerau M.J., Walsh J.L., Wong KF K., Salazar-Gomez A.F., Harrell P.G., Sampson A.L., Cimenser A. (2023). Electroencephalogram signatures of loss and recovery of consciousness from propofol. Proc. Natl. Acad. Sci. USA.

[34] Brown E.N., Purdon P.L., Van Dort C.J. (2011). General Anesthesia and Altered States of Arousal: A Systems Neuroscience Analysis. Annu. Rev. Neurosci..

[35] Seamans J. (2008). Losing inhibition with ketamine. Nat. Chem. Biol..

[36] Homayoun H., Moghaddam B. (2007). NMDA Receptor Hypofunction Produces Opposite Effects on Prefrontal Cortex Interneurons and Pyramidal Neurons. J. Neurosci..

[37] Domino E.F., Warner D.S. (2010). Taming the Ketamine Tiger. Anesthesiology.

[38] Akeju O., Song A.H., Hamilos A.E., Pavone K.J., Flores F.J., Brown E.N., Purdon P.L. (2016). Electroencephalogram signatures of ketamine anesthesia-induced unconsciousness. Clin. Neurophysiol..

[39] Bojak I., Day H.C., Liley D.T.J. (2013). Ketamine, Propofol, and the EEG: A Neural Field Analysis of HCN1-Mediated Interactions. Front. Comput. Neurosci..

[40] Ko J.C., Raffe M.R., Knels O., Inoue T., Weil A.B. (2009). Analgesia, Sedation and Anesthesia: Making the Switch from Medetomidine to Dexmedetomidine. Compend. Contin. Educ. Vet..

[41] Ko J.C.H., Fox S.M., Mandsager R.E. (2000). Sedative and cardiorespiratory effects of medetomidine, medetomidine-butorphanol, and medetomidine-ketamine in dogs. J. Am. Vet. Med. Assoc..

